# Together we are strong! Collaboration between clinicians and engineers as an enabler for better diagnosis and therapy of atrial arrhythmias

**DOI:** 10.1007/s11517-023-02788-0

**Published:** 2023-02-07

**Authors:** Axel Loewe, Armin Luik, Roberto Sassi, Pablo Laguna

**Affiliations:** 1 Institute of Biomedical Engineering, Karlsruhe Institute of Technology (KIT), Karlsruhe, Germany; 2 Medizinische Klinik IV, Städtisches Klinikum Karlsruhe, Karlsruhe, Germany; 3 Università Degli Studi Di Milano, Milan, Italy; 4 BSICoS Group, Aragón Institute of Engineering Research (I3A), University of Zaragoza, Zaragoza, Spain; 5 Centro de Investigación Biomédica en Red en Bioingeniería, Biomateriales Y Nanomedicina (CIBER-BBN), Zaragoza, Spain

The first electrocardiogram showing atrial fibrillation was published by Willem Einthoven already in 1906; also electrograms recorded within the atrial cavities in humans were described more than 70 years ago [1]. Since then, the marked improvements of early and accurate diagnosis of atrial arrhythmias, tailored treatment, and long-term success were in large parts driven by technological progress in signal acquisition and processing hardware as well as analysis algorithms in close interaction with clinical users and clinical studies evaluating outcome improvement. Olaf Dössel from Karlsruhe Institute of Technology (KIT) and Claus Schmitt from Städtisches Klinikum Karlsruhe established such close interaction between their teams and realized the synergistic potential very quickly. This essential role of close collaboration between engineers and clinicians, paired with the remaining great challenges, such as the treatment of persistent atrial fibrillation, led to the establishment of the Atrial Signals conference in 2015. After editions in Valencia and Bordeaux, Atrial Signals returned to Karlsruhe in autumn 2021 [2] with more than 70 oral presentations and panel discussions from leaders in the field, both clinical and technical. This event also marked the retirement of both Olaf Dössel and Claus Schmitt.

Olaf Dössel’s innovations in the field of computational cardiology are numerous. In more than 25 years of research, his main contributions were in signal processing of electrograms and electrocardiograms [3], as well as in computational modeling and simulation of the heart with a particular focus on tissue level [4] and organ level/ECG applications [5]. He served on several editorial boards including this Journal, was president of the World Congress on Medical Physics and Biomedical Engineering in 2009, is a long-standing member of multiple academies of science, the board of directors of the Computing in Cardiology Conference, and a fellow of the International Union for Physical and Engineering Sciences in Medicine (IUPESM), the International Academy of Medical and Biological Engineering (IAMBE), the European Alliance of Medical and Biological Engineering and Science (EAMBES) and the German Society for Biomedical Engineering (DGBMT). Besides his direct scientific track record of several hundred publications, another important part of his legacy are more than 10,000 students that learned the basics of electrical engineering in his lectures and more than 60 early career researchers that completed a PhD under his guidance. The strong commitment and never-ending excitement to develop engineering approaches to a point where they can be applied in a clinical research setting paired with a lot of room to grow and develop for everyone is remarkable.

Claus Schmitt is a pioneer in invasive electrophysiology and cardiology. Inspired and shaped by a research fellowship in electrophysiology at the University of Pennsylvania under the direction of Marc E. Josephson in the late 1990s, he was captivated by the idea of understanding cardiac arrhythmias in detail and being able to cure them by catheter ablation. He was head of the electrophysiology department at the German Heart Center in Munich for more than 10 years and established the clinical electrophysiology as well as implantation of pacemakers and defibrillators there. He gained a significant international reputation through constant innovation and the use of new technologies up to the commissioning of one of the first stereotactic ablation systems worldwide. In more than one hundred publications, many congress papers, and several books, he openly addressed problems and challenges. He accompanied several randomized studies and was thus able to improve existing techniques. His educational books on intracardiac electrophysiology and catheter ablation of cardiac arrhythmias have guided generations of electrophysiologists during their training. However, his primary fascination from beginning to end was the ECG and the interpretation of intracardiac electrograms. With the move to Karlsruhe and the cooperation with Olaf Dössel, computer models and algorithms entered the electrophysiology laboratory and raised the interpretation of electrograms to a new level. With the common goal of developing an individualized approach for the treatment of atrial fibrillation, Claus Schmitt and Olaf Dössel started a unique close cooperation between clinicians and engineers. Claus Schmitt’s never-ending drive to steadily improve methods and his courage to use them for the benefit of his patients, coupled with constant encouragement and challenge of individual team members is remarkable.

This special issue brings together seven articles summarizing some of the work presented at the fourth Atrial Signals conference in 2021 in Karlsruhe. Together with other recent special issues on atrial arrhythmias and technology for their diagnosis and treatment [6–8], they provide a good overview of the current state of the art and recent progress.

Two review articles summarize the state of the art, recent progress and open challenges in the fields of atrial conduction velocity mapping (Coveney et al. [9]) and application of electrocardiographic imaging (ECGI) in the atria (Hernández-Romero et al. [10]).

Five original articles present new methods, results and tools for better understanding, diagnosis, and treatment of atrial arrhythmias.

In a computational basic research study, Elliott et al. [11] investigate the effect of cellular heterogeneity on atrial repolarization and identified differences between the left and the right atrium. Riccio et al. [12] leverage multipolar electrograms to identify fibrotic atrial substrates based on dominant-to-remaining eigenvalues of unipolar electrograms of neighboring electrodes. After benchmarking their method on synthetic electrograms, they demonstrate feasibility in measured electrograms.

Heida et al. [13] use epicardial mapping data from 34 patients to better characterize the supervulnerable period after electrical cardioversion of atrial fibrillation. They found no significant differences in biatrial conduction differences between atrial fibrillation patients who were acutely cardioverted and those who already were in sinus rhythm for longer.

Dang et al. [14] use clinical data to correlate the information content of dominant frequency and slow conduction zone maps during atrial fibrillation. They found no significant correlation between the two types of maps in their cohort of 19 patients.

Finally, van Nieuwenhuyse et al. [15] present Directed-Graph-Mapping as a new tool to automatically analyze and characterize cardiac excitation patterns based on local activation time maps. They present a range of application scenarios including atrial tachycardia and fibrillation.

Despite the remarkable progress in the field of atrial arrhythmia diagnosis and treatment, many challenges remain open. Olaf Dössel and Claus Schmitt have demonstrated how close collaboration between engineers and clinicians can enable new solutions and their implementation. We believe that this example can be an excellent motivation and driver for future endeavors and we wish them all the best for their retirement.
